# What surgeons should know about emergency operation for COVID-19 confirmed patients: A case report

**DOI:** 10.1016/j.ijscr.2020.10.137

**Published:** 2020-11-04

**Authors:** Dongkyu Oh, Yu Min Kang, Jin Yong Choi, Wang Jun Lee

**Affiliations:** a Department of General Surgery, Myongji Hospital, 697-1 Hwajung-dong, Deokyang-gu, Goyang-si, Gyeonggi-do, Republic of Korea; b Department of Infectious Diseases, Myongji Hospital, 697-1 Hwajung-dong, Deokyang-gu, Goyang-si, Gyeonggi-do, Republic of Korea

**Keywords:** COVID-19, SARS CoV-2, Emergency surgery, Negative-pressure operating room, Case report

## Abstract

**Introduction:**

This case is shared to reiterate and confirm the principles of ensuring the safety of the surgical team caring for COVID-19-confirmed patients, thus, preventing the spread of infection within the hospital.

**Case presentation:**

A 54-year-old male, COVID-19-confirmed patient complaining of abdominal pain since two days prior was transferred to our hospital. Perforated appendicitis with a periappendiceal abscess was diagnosed by computed tomography. Laparoscopic appendectomy was performed in a negative-pressure operating room. The surgical team wore enhanced personal protective equipment. Electrocautery was not used during surgery and no other special instruments were applied to reduce aerosol generation. No special instruments or filters were used for the removal of intra-abdominal gas. The operation was completed successfully and no immediate surgical complications occurred. The patient advanced to a normal diet on the 4th postoperative day. The patient was treated with antibiotics for bacteremia and antiviral therapy for underlying pneumonia in the setting of COVID-19 with most symptoms dissipating by the 7th postoperative day. The patient was discharged on the 30th postoperative day without any complications.

**Discussion:**

A well-designed manual, a well-trained surgical team, and a negative-pressure operating room are essential for safe laparoscopic appendectomies in COVID-19 patients.

**Conclusion:**

When surgery is performed in a negative-pressure operating room by a well-trained surgical team, a laparoscopic appendectomy can be successfully performed under the principles of obtaining optimum clinical outcomes while faithfully ensuring the safety of healthcare providers and the hospital environment.

## Highlights

• Laparoscopic appendectomy for COVID-19 patients can be safely performed without the risk of further infection spread.
• A negative-pressure operating room and proper personal protective equipment are essential for safer laparoscopic procedures.
• Sustainable institutional strategies and a well-trained multidisciplinary team are mandatory to overcome the limitations of surgical procedures during the COVID-19 pandemic.

## 1 Introduction

Various guidelines on how to perform emergency surgery during the COVID-19 era are currently evident throughout the literature [1]. The principle of preventing the spread of infection among surgical teams and the hospital environment without adversely affecting the patients’ clinical outcomes is the commonality of these guidelines [2]. We intend to share a case of laparoscopic appendectomy performed on a COVID-19 patient as verification that these principles can be faithfully applied and executed within the surgical field.

The hospital involved was a 550-bed tertiary referral hospital, which operates a Level 1 emergency center and a 12-bed negative-pressure isolation ward including five negative-pressure isolation ICU beds [3]. It operates 13 operating rooms including four negative-pressure rooms and performs more than 400 emergency appendectomies per year.

This case report was drafted according to the SCARE Guidelines 2018 [4].

## 2 Case presentation

A 54-year-old male patient was transferred with diagnosed acute perforated appendicitis. The patient was admitted to a public hospital after being confirmed for COVID-19 by real-time polymerase chain reaction (RT-PCR) analysis four days before the transfer. The patient had no complaints including respiratory symptoms at the time of admission. Lower abdominal pain and diarrhea began two days prior to transfer, and he was diagnosed with perforated appendicitis on a non-contrast abdominal computed tomography (CT) scan. The public hospital mainly treated mild COVID-19 cases, and because it was not suitable for performing the emergency surgery, the patient was transferred to our hospital facility, dedicated to treating complicated COVID-19 cases and equipped with a negative-pressure operating room.

The patient was taking anti-hypertensive medication that did not contain anticoagulants and had no significant surgical, family, or social history. The patient was an immigrant construction worker.

The patient complained of only abdominal pain and did not report any respiratory symptoms such as coughing or sputum. The patient was 176 cm tall and 75 Kg with a body mass index (BMI) of 24.2. The physical examination was significant for tenderness and rebound tenderness throughout the lower abdomen. The vital signs showed 80/52 mmHg blood pressure, a pulse of 98 beats/min, a respiratory rate was 20 times/min, a body temperature of 36.2 OC, and 96 % oxygen saturation on room air.

**Fig. 1:**
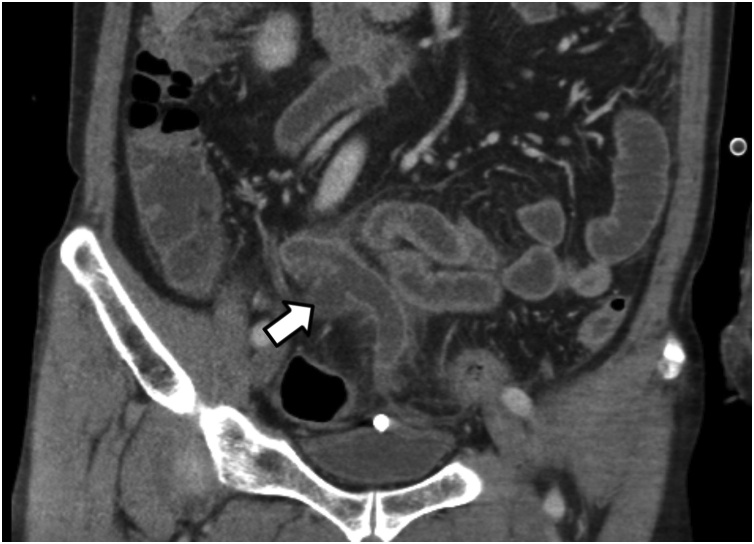
Preoperative CT imaging findings. An enlarged, perforated appendix with surrounding abscess indicated by a white arrow.

Laboratory investigations revealed a white blood cell (WBC) count of 15.3 × 10³/㎕ and a C-reactive protein (CRP) value of 18.30 mg/dL. The other laboratory findings were not specific. Since information from the non-contrast CT scan that accompanied the patient was limited, a contrast-enhanced abdominal CT scan was ordered to establish a more precise surgical plan. The CT scan showed perforated acute appendicitis with a periappendiceal abscess and widespread pelvic peritonitis (Fig. 1). There was no evidence of pneumonia on chest CT imaging.

Based on the above findings, upon written informed consent for the procedure and notification of the risks and benefits, a faculty surgeon with nine years of experience performed an emergency laparoscopic appendectomy. The CT scan findings showed inflammatory processes spread throughout the pelvic cavity, so it was felt that laparoscopic appendectomy was less invasive and could be completed within a short time, and thus, considered more favorable for the treatment outcome without the risk of further infection spread.

**Fig. 2:**
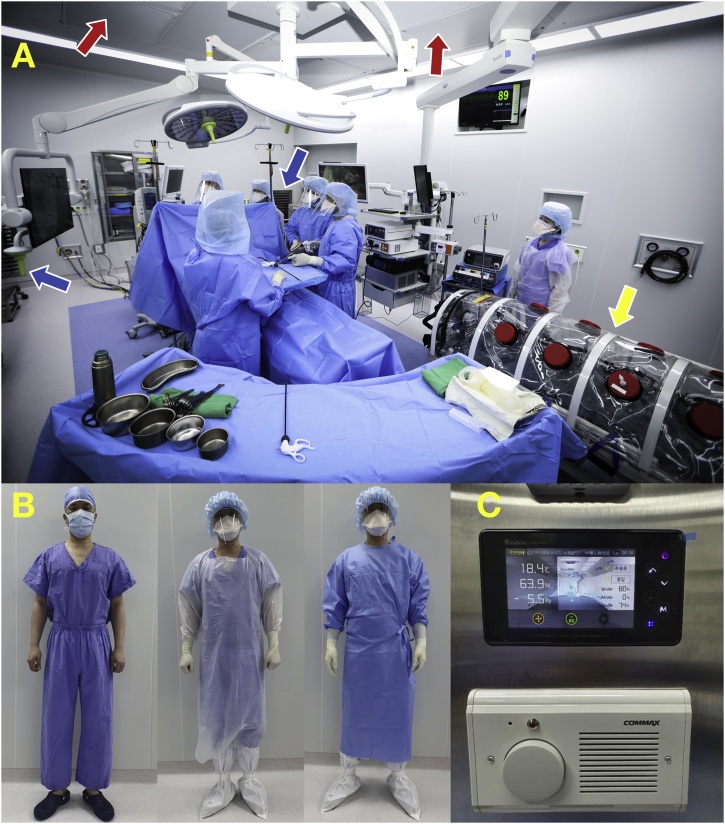
A) View of the entire negative-pressure operating room. The surgical team is wearing enhanced personal protective equipment and a negative-pressure cart is shown (yellow arrow). Airflow was maintained from the ceiling perforated supply diffuser (red arrow) to the exhaust grille (blue arrow) installed at the bottom of the four corners of the operating room. B) Surgical suit, an isolation gown, and enhanced personal protective equipment. C) Negative-pressure status display panel and intercom installed outside the entrance of the negative-pressure operating room.

The patient entered the operating room on a negative-pressure cart, which was placed in the operating room (OR) during the surgery (Fig. 2).

General anesthesia was performed through endotracheal intubation using a video laryngoscope by an anesthesiologist wearing a Level 4 protective suit. The operation was initiated by the surgical team wearing enhanced personal protective equipment (PEE). The enhanced PPE included an isolation gown, surgical gown, head cover, face shield, two pairs of surgical gloves, shoe covers, and an N95 mask [5] (Fig. 2).

A 12-mm port was applied to the umbilicus, and a 5 mm port was applied to the upper pubis and the left para-midline. Pneumoperitoneum was formed to 14 mmHg abdominal pressure at a CO_2_ flow rate of 40 L/min. A closed-circuit smoke evacuation system was not used. The perforated appendix and the spread of inflammation to the surrounding organs were observed, and suppurative fluid was accumulated in the pelvic cavity. Electrocautery was not used during surgery. An ultrasonic scalpel for dissection, a plastic clip for vascular ligation, and an endo-loop for stump ligation of the appendix were all used [6]. Disposable devices were used whenever possible. The abdominal cavity was washed several times, and a self-retained negative-pressure silicone drainage tube was replaced. When the intra-abdominal gas was removed after the procedure, the valve was gradually opened so that the gas did not dissipate too suddenly, and no special instruments or filters were used in this step.

Personnel exiting the OR disposed of their PPE in the OR under supervision and showered immediately thereafter. Dedicated well-identifiable containers for infectious-risk waste were used.

The patient was monitored in a negative-pressure cart in that OR until the vital signs stabilized and the patient was fully conscious before being transferred to a negative-pressure isolation ward. The patient was subsequently transferred to the department of infectious disease for postoperative care.

The operation lasted 45 min, anesthesia was applied for 60 min, and the operating room retention time was 90 min.

The operating room was maintained under negative-pressure air conditioning for two hours after the patient was moved out. Following three hours of fumigating disinfectant, surface disinfection with diluted chlorine bleach (500 ppm) was applied. Five hours after the operation was completed, the operating room was ready for use again. The entire process of patient transfer, preparation of the operating room, the surgical procedure, and postoperative disinfection was monitored and recorded by an infection specialist nurse.

All participating members of the surgical team were monitored actively for two weeks. There were no signs of medical staff infection nor the spread of infection within the operating room.

On the 4th postoperative day, the patient started a normal diet. Pneumonia was found on a chest CT scan, prompted by subjective complaints of coughing symptoms, and hydroxychloroquine therapy was started [7]. The drainage tube was removed on the 5th postoperative day. Fever, likely due to *Staphylococcus capitis* infection detected on blood cultures, persisted until the 6th postoperative day with resolution following antibiotic therapy. On the 7th postoperative day, the respiratory and abdominal symptoms improved, the WBC count normalized, and the CRP level decreased to 1.43 mg/dL.

No postoperative complications were observed, and after two consecutive negative results for COVID-19 by RT-PCR, the patient was discharged on the 30th postoperative day. Follow-up was not possible because the patient returned to his home country immediately after discharge.

## 3 Discussion

Before this case, we experienced eight cases of emergency laparoscopic appendectomies performed before COVID-19 tests were confirmed. As a hospital dedicated to infectious diseases, a multidisciplinary team had developed an instruction manual for emergency surgery for confirmed COVID-19 patients. The manual was improved through training and debriefing via several simulations [3,8]. It is believed that the series of preparatory courses centered on multidisciplinary teams and surgical experiences served as the basis for a successful laparoscopic appendectomy for this confirmed patient.

One of the concerns in laparoscopic surgery for COVID-19-confirmed patients is the proper care of aerosols containing viruses that could be produced by these patients. In this case, no electrocautery was used, and no special device was used for intra-peritoneal gas leaking out during surgery or for intra-peritoneal gas discharge following surgery [9]. We believe that a negative-pressure OR addresses many of these concerns [10]. The negative pressure inside the OR was maintained at a negative pressure of 4.7 Pa or greater, and the air introduced from the perforated supply diffuser from the ceiling was discharged through an exhaust grill located at a height of 110 cm from the floor at all four corners of the OR [11] (Fig. 2). Airflow in these rooms reached 14–18 air exchanges per hour. Therefore, airflow from the operating table to the floor was maintained. This significantly minimized the possibility of inhaling harmful gas generated from the patient without additional devices or equipment [12].

There is a risk of infection to the surgical team during surgery, but there is a higher probability of contamination, especially when PPEs are removed after surgery [13]. Therefore, the surgical team must vigilantly observe whether there are any signs of contamination. In particular, it is essential to supervise and monitor the entire operation process, exiting of the surgical team, and the postoperative disinfection tasks by the infection specialist nurse.

In this case report, there was a limit to reviewing the overall considerations for laparoscopic appendectomy for patients with confirmed COVID-19. Therefore, follow-up analysis and research are warranted for all surgical cases suspected of a positive diagnosis as well as the confirmed patients.

## 4 Conclusion

When surgery is performed in a negative-pressure OR by a well-trained surgical team, a laparoscopic appendectomy can be performed under the principle of obtaining optimum clinical outcomes while faithfully ensuring the safety of healthcare providers and the hospital environment.

## Declaration of Competing Interest

The authors report no declarations of interest.

## Funding

No funding involved in this case study.

## Ethical approval

On the basis of this being a case report, the Institutional Review Board of the Myongji hospital does not mandate that ethical approval is required. Thus, this case report is exempt from the Institutional Review Board Approval process (IRB approval number MHJ 2020-10-037).

## Consent

Written informed consent was not obtained from the patient for publication of this case report because the patent was leaving the country. A letter stating that the hospital president will take full responsibility for the patient's damage caused by publication is available for review by the Editor-in-Chief of this journal on request.

## Author’s contribution

Major contributors to writing the manuscript: Dongkyu Oh and Yu Min Kang.

Searching the literature, contributor to writing and reviewing the manuscript: Jin Yong Choi.

Reviewing the manuscript for important intellectual content: Yu Min Kang.

Study concept and final review: Wang Jun Lee.

All authors read and approved the final manuscript.

## Registration of research studies

NA.

## Guarantor

Wang Jun Lee, M.D., PhD.

## Provenance and peer review

Not commissioned, externally peer-reviewed.
